# Perilipin-2 Modulates Lipid Absorption and Microbiome Responses in the Mouse Intestine

**DOI:** 10.1371/journal.pone.0131944

**Published:** 2015-07-06

**Authors:** Daniel N. Frank, Elise S. Bales, Jenifer Monks, Matthew J. Jackman, Paul S. MacLean, Diana Ir, Charles E. Robertson, David J. Orlicky, James L. McManaman

**Affiliations:** 1 Division of Infectious Disease, University of Colorado School of Medicine, Aurora, Colorado, United States of America; 2 Microbiome Research Consortium, University of Colorado School of Medicine, Aurora, Colorado, United States of America; 3 Division of Basic Reproductive Sciences, University of Colorado School of Medicine, Aurora, Colorado, United States of America; 4 Division of Endocrinology and Metabolism, University of Colorado School of Medicine, Aurora, Colorado, United States of America; 5 Department of Pathology, University of Colorado School of Medicine, Aurora, Colorado, United States of America; 6 The Center for Human Nutrition, University of Colorado School of Medicine, Aurora, Colorado, United States of America; National Institute of Agronomic Research, FRANCE

## Abstract

Obesity and its co-morbidities, such as fatty liver disease, are increasingly prevalent worldwide health problems. Intestinal microorganisms have emerged as critical factors linking diet to host physiology and metabolic function, particularly in the context of lipid homeostasis. We previously demonstrated that deletion of the cytoplasmic lipid drop (CLD) protein Perilipin-2 (Plin2) in mice largely abrogates long-term deleterious effects of a high fat (HF) diet. Here we test the hypotheses that Plin2 function impacts the earliest steps of HF diet-mediated pathogenesis as well as the dynamics of diet-associated changes in gut microbiome diversity and function. WT and perilipin-2 null mice raised on a standard chow diet were randomized to either low fat (LF) or HF diets. After four days, animals were assessed for changes in physiological (body weight, energy balance, and fecal triglyceride levels), histochemical (enterocyte CLD content), and fecal microbiome parameters. Plin2-null mice had significantly lower respiratory exchange ratios, diminished frequencies of enterocyte CLDs, and increased fecal triglyceride levels compared with WT mice. Microbiome analyses, employing both 16S rRNA profiling and metagenomic deep sequencing, indicated that dietary fat content and Plin2 genotype were significantly and independently associated with gut microbiome composition, diversity, and functional differences. These data demonstrate that Plin2 modulates rapid effects of diet on fecal lipid levels, enterocyte CLD contents, and fuel utilization properties of mice that correlate with structural and functional differences in their gut microbial communities. Collectively, the data provide evidence of Plin2 regulated intestinal lipid uptake, which contributes to rapid changes in the gut microbial communities implicated in diet-induced obesity.

## Introduction

Obesity, resulting from increased calorie consumption and decreased energy expenditure, remains a major world-wide public health concern for both children and adults [1], through its strong etiological links to several metabolic complications, including metabolic syndrome, Type-2 diabetes and non-alcoholic fatty liver disease (NAFLD) [2]. Intestinal bacteria have been causally implicated in human metabolic diseases, including obesity, type-2 diabetes and NAFLD [3], as well as in certain cancers [4]. Moreover, intestinal bacteria influence fat storage in adipose and other organs in mice [5]. A prominent hypothesis linking intestinal dysbiosis to obesity is that members of the bacterial phylum Firmicutes are more efficient than other gut-associated microbial taxa in converting indigestible foods to nutrients that are useable by the host; expansion of these Firmicutes in the gut is thus thought to increase the flux of calories into the host [6]. In turn, dietary fat composition and content are primary determinants of intestinal microbiome composition in fish, rodents and humans [7,8]. These observations suggest that intestinal bacteria play coordinating roles in managing the nutrient utilization and calorie deposition of their hosts, and that disrupting these functions, by altering the composition and/or metabolic properties of the intestinal bacterial community, may contribute to the host’s susceptibility to metabolic disease.

Due to its essential role in the uptake and transport of nutrients in the gut, the intestinal epithelium is likely to be a primary target of bacteria-host interactions that influence host metabolism and metabolic disease risk. Detailed information about how diet and gut microbiota influence the physiological, metabolic, and immunological properties of intestinal epithelial cells is limited. However, both diet and intestinal microbiome composition influence nutrient uptake and metabolic functions of the intestine [7,9]. Combined with established influences of diet on the composition and metabolic properties of intestinal microorganisms [7], these observations suggest that nutrient uptake is regulated by cooperative effects of diet on the properties of the intestinal epithelium and gut microbial cells.

Cytoplasmic lipid droplets (CLD) are organelle-like structures that are increasingly recognized as important homeostatic regulators of cellular and tissue lipid metabolism; functioning as energy reservoirs, as a mechanism for limiting the levels of bioactive, and potentially toxic, free fatty acids, and in lipid trafficking within cells [10]. Members of the perilipin (PLIN) family (perilipin-1, Plin1; perilipin-2, Plin2, ADRP, ADPH, ADFP; perilipin-3, Plin3, TIP47; perilipin-4, Plin4, S3-12; perilipin-5, Plin5, OXPAT, LSDP5) are CLD scaffold proteins that serve as critical determinants of CLD formation and cellular lipid metabolism within mammalian cells [11]. Under normal conditions, Plin2 is the most abundantly expressed PLIN family member in livers of humans [12] and mice [13]. Plin2 is also highly expressed in the human [14] and murine [15] small intestine. Our published studies have documented that Plin2 is required in the long term for high fat (HF) diet-induced obesity and fatty liver disease in mice [13]. However, specific effects of Plin2 on intestinal lipid properties have not been established, and details about the physiological function(s) of CLD in intestinal epithelial cells are only beginning to emerge [16–18]. Here we investigate whether the actions of Plin2 involve effects on intestinal lipid absorption and intestinal microbiome properties at the outset of exposure to HF- and LF-diets.

## Materials and Methods

### Animal procedures

Eight-week old male C57BL/6 (WT) and perilipin-2 null (Plin2-null) mice were used for all studies. The generation and characterization of Plin2-null mice have been described in detail previously [13]. WT mice used in this study were obtained from a breeding colony maintained at the University of Colorado School of Medicine’s Center for Comparative Medicine and housed in the same room as Plin2-null mice. All mice were fed standard mouse chow (2020X, Harlan Laboratories, 16% fat calories, 24% protein calories and 60% carbohydrate calories) *ad libitum* from weaning to 8 weeks of age, at which time they were housed individually in a metabolic monitoring system at 30°C, the thermoneutral temperature of mice [19], for measurements of energy balance (intake and expenditure), the respiratory exchange ratio (RER), and activity levels (Columbus 8M Oxymax) [20]. Following a 3-day adjustment period, the mice were fed nutritionally balanced high fat (HFD, 60% fat calories, 20% protein calories, 20% carbohydrate calories, D12492) or low fat (LFD, 10% fat calories, 20% protein calories, 70% carbohydrate calories, D12450B) diets from Research Diets Inc. (New Brunswick, NJ) *ad libitum* for 4 days. Feces were collected daily. Body weights and compositions were measured at the beginning and end of dietary treatments. Body compositions were determined by quantitative magnetic resonance (QMR; EchoMRI-900 Whole Body Composition Analyzer; Echo Medical Systems, Houston, TX). Tissues were harvested from chambered housed mice during their light cycle after fasting for approximately 8hr.

For fasting-feeding experiments, mice were fed the HFD for 4 days, fasted for 24h then refed with the 60% fat calorie diet for 2 hours. At the termination of each experiment, mice were injected with sodium pentobarbital to induce anesthesia and perfused with paraformaldehyde [21] or euthanized by exsanguination. The University of Colorado School of Medicine Institutional Animal Care and Use Committee approved all procedures.

### Histological, immunofluorescence and BODIPY staining

Livers and intestines were processed for hematoxylin and eosin (H&E) staining and immunofluorescence microscopy as described [13]. Plin2 and Plin3 were detected by reaction with guinea pig anti-Plin2 (1:1000) and rabbit anti-Plin3 (1:1000) and visualized using Alexafluor 488 and Alexafluor 594 secondary antibodies respectively [13]. For BODIPY (boron-dipyrromethene) staining, intestines from paraformaldehyde-perfused animals were sectioned at 10 um, collected onto Cell-Tak coated coverslips, and vapor-fixed with paraformaldehyde for 20 min before being gently rehydrated with PBS. Auto-fluorescence was quenched with 2mg/ml glycine for 10 min. Sections of the intestine were rinsed with PBS and stained with BODIPY 493/503 at a final concentration of 30 ug/ml to identify CLD and Alexafluor 488-labeled wheat germ agglutinin (Alexafluor-WGA) to identify villi surfaces. Coverslips were mounted in PBS and imaged within 3 days. Confocal imaging of BODIPY 493/503 and Alexafluor-WGA performed on a 3I Marianas Inverted Spinning Disk Confocal system. Relative neutral lipid levels were estimated by quantifying BODIPY fluorescence in individual villi from 8–10 sections, containing 4–6 villi per section, from the jejunum and ileum. Total fluorescence area in each villi were normalized to the number of nuclei using masking functions built into SlideBook Image Analysis Software (Intelligent Imaging Innovations, Inc., Denver, CO) [22] to estimate neutral lipid levels per cell. All images were processed by Photoshop (Adobe Systems Inc., Mountain View, CA).

### Fecal lipids

Triglycerides (TG) in fecal lipid extract were determined using commercially available kits [22] and quantified based on reference standards. TG in feces were normalized to fecal weight.

### 16S rRNA Analysis

Bacterial profiles were determined by broad-range amplification and sequence analysis of 16S rRNA genes following our previously described methods [23,24]. DNA extractions from mouse feces were performed using the PowerFecal DNA Isolation kit (MoBio Laboratories, Inc., Carlsbad, CA) according to the manufacturer’s protocols. Bacterial 16S rRNA gene copies in each sample were enumerated by quantitative PCR (QPCR) [25] and samples normalized to ~10^6^ templates/microliter. PCR amplicons were generated using barcoded primers [26] that target approximately 300 b.p. of the V1V2 variable region of the 16S rRNA gene (primers 27FYM [27] and 338R [28]). Paired-end, multiplexed sequencing of 16S amplicon pools was performed as previously described[24,29] on an Illumina MiSeq platform (Illumina Inc, San Diego, CA, USA) using the 600-cycle MiSeq Reagent Kit v3 [24]. All 16S rRNA and metagenomic sequences were deposited into the NCBI short-read archive under project number PRJNA283468.

Illumina Miseq paired-end sequences were demultiplexed, quality-filtered, and assembled as previously described [24,29–31]. Potential chimeras identified with Uchime (usearch6.0.203_i86linux32) [32] using the Schloss [33] Silva reference database were removed from subsequent analyses. Assembled sequences were aligned and classified with SINA (1.2.11) [34] using the 629,124 bacterial sequences in Silva 111Ref [35] as reference configured to yield the Silva taxonomy. Operational taxonomic units (OTUs) were produced by clustering sequences with identical taxonomic assignments. A median of 166,374 high-quality 16S rRNA sequences were generated per fecal specimen [IQR (interquartile range) 151,286–176,661]. All datasets had Good’s coverage indices greater than 99.9%, indicating that the depth of sequencing was sufficient to fully describe the biodiversity of the samples.

Community-wide differences in microbiome composition (i.e. beta diversity) between experimental groups were assessed using a non-parametric multivariate analysis of variance test (PERMANOVA) [36] implemented by the *adonis* function of the R package *vegan*, [37] with beta-diversity measured using Morisita-Horn similarities; P-values were estimated following 100,000 replicate label permutations. Specific microbial groups (species/genera/phyla) that differed between experimental groups were identified by Wilcoxon rank-sum test with continuity correction and exact P-values. P-values for both the PERMANOVA and Wilcoxon tests were corrected for multiple comparisons using Benjamini and Hochberg’s false discovery rate method [38]. Standard ecological alpha-biodiversity indices (e.g., S_obs_, Shannon diversity [39,40]), inferred by rarefaction and bootstrap analysis with 1000 replicate re-samplings, were assessed by ANOVA using diet and genotype as predictor variables. Differences between pairs of genotype/diet groups were assessed by Tukey’s Honest Significant Difference tests. Principal components analysis (PCA) used the R prcomp command to analyze a covariance matrix of center-log transformed [41] sequence count data, classified to the genus-level. In the plots presented, PC1, PC2, and PC3 accounted for 35.3%, 26.8%, and 15.7% of the total variance in the dataset (77.8% total). Associations of principal component axes 1 and 2 scores with genotype and diet were evaluated by Welch Two-Sample two-sided t-test. Statistical analyses were assessed at α = 0.05. The software package Explicit (v2.9.3)[42] was used for calculation of alpha-biodiversity indices, display, Wilcoxon rank-sum tests of OTU distributions between categories, and figure generation of results. The R statistical package (v3.0.3)[43] was used for all other analysis and figure generation.

### Metagenomic Analysis

Bulk fecal DNA samples prepared for 16S PCR were subjected to multiplexed shotgun sequencing using the Nextera XT kit (Illumina Inc, San Diego, CA, USA) and the 600-cycle MiSeq Reagent Kit v3 (Illumina Inc, San Diego, CA, USA). Raw, paired-end reads were trimmed of poor-quality bases at 5’ and 3’ ends (by excising bases in a 10 nucleotide sliding window with mean phred Q<15 [44]), assembled using FLASH [45], and a total of 10,548,626 high-quality merged reads uploaded to the metagenomic RAST server (MG-RAST; http://metagenomics.anl.gov; accessed Nov 2013 –Aug 2014) for automated sequence classification and analysis [46]. Murine sequences were identified and culled by comparison to the *Mus musculus* NCBI v37 reference genome and the remaining sequences were annotated using the MD5 nonredundant database [47]. Results are presented for annotations against the KEGG (Kyoto Encyclopedia of Genes and Genomes) ortholog (KO) hierarchy [48]; SEED [49] and COG [50] annotations generated qualitatively similar results (data not shown). Sequence annotations were downloaded and analyzed using the R package “matR” v1.0.0) http://www.mcs.anl.gov/~braithwaite/library/matR/html/matR-package.html). Datasets were log-scaled and centered using the matR “normalize” command and KO functional annotations that differed in frequency between experimental groups identified through the matR “sigtest” function using a Kruskal-Wallis test. Because of the pilot nature of this study, p-values were not adjusted to control for false discovery rate. Heatmaps were imported into Adobe Illustrator to add annotations. Higher and lower level KO categories were mapped after downloading the KO ontology using the MG-RAST API (http://api.metagenomics.anl.gov/api.html; accessed August 2014).

All authors had access to all the data and have reviewed and approved the final manuscript.

## Results

### Effects of Plin2 deletion on metabolic responses to low fat (LF) and high fat (HF) diet feeding

**Fig 1:**
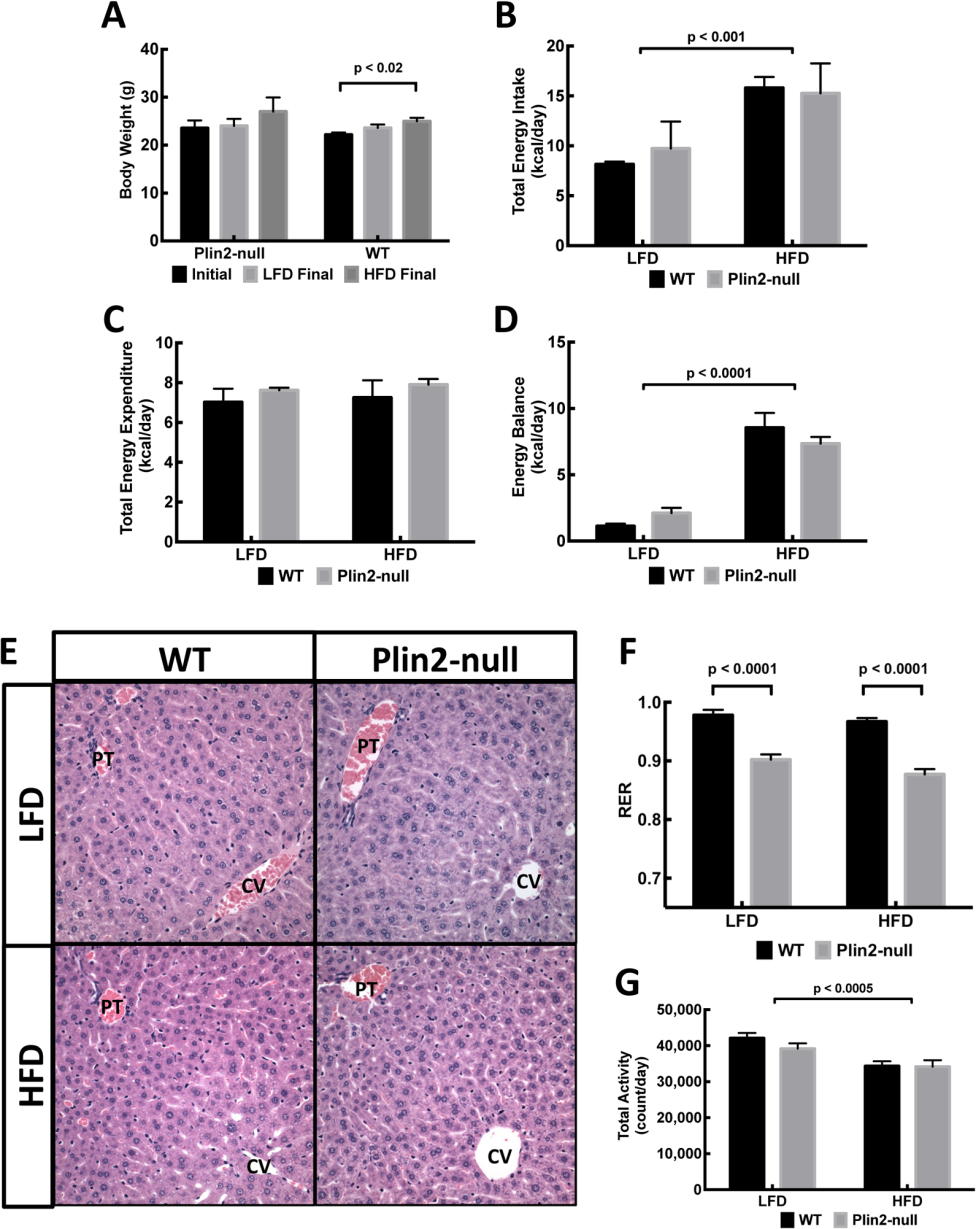
Metabolic effects of four-day LF and HF feeding on WT and Plin2-null mice. (A) Body weights of eight-week-old WT and Plin2-null fed the chow diet for 5 weeks (Initial) and after four days of LF (LFD Final) or HF (HFD Final) diet feeding. (B-D) Energy intake (B), energy expenditures (C) and energy balance (D) properties. (E) Representative H&E stained liver sections of LF and HF fed WT or Plin2-null mice at 200X magnification. PT, portal triad. CV, central vein. (F,G) Average respiratory exchange ratios (RER) (F), and total activities (G) of WT and Pln2-null mice fed LF or HF diets. Data were analyzed by 2-way ANOVA examining the effects of diet, genotype, and their interaction. Statistical significance of diet on each parameter is indicated in the respective figures. All values are means ± SEM, N = 4 per group.

Our previous studies have documented that Plin2 expression is required for long-term weight gain, increased adiposity, and fatty liver formation in mice fed HF diets [13]. These initial studies suggested that the effects of Plin2 on these properties were mediated in part by its actions on energy intake and activity levels of HF fed mice. Because, HF diet feeding has also been shown to have acute effects on energy intake and metabolic properties of mice [51,52], we were interested in determining how Plin2 affects short-term responses to HF diet feeding. We addressed this question by placing 8 week-old congenic WT (C57B/6) and Plin2-null male mice, which had been maintained since weaning on a mouse chow diet (16% kcal fat, 24% kcal protein, 60% kcal carbohydrate), in metabolic chambers and feeding them *ad libitum* with calorically equivalent LF (10% kcal fat, 20% kcal protein, 70% kcal carbohydrate) or HF (60% kcal fat, 20% kcal protein and 20% kcal carbohydrate) diets (Table 1) for four days. Data in Fig 1A show that during this period body weights of Plin2-null mice did not change significantly on either diet. The body weights of WT mice did not change on the LF diet, but they increased about 10% (p<0.02) on the HF diet.

**Table 1:** Caloric Content of Low- and High-Fat Diets.

	Low Fat (10 kcal% fat) Diet	High Fat (60 kcal% fat) Diet
Component	kcal	kcal
Casein	**800**	**800**
L-Cysteine	**12**	**12**
Corn Starch	**1260**	**0**
Maltodextrin	**140**	**500**
Sucrose	**1400**	**275**
Soybean Oil	**225**	**225**
Lard	**180**	**2205**
Vitamin mix	**40**	**40**
Total	**4057**	**4057**

HF fed WT and Plin2-null mice consumed significantly more calories than their LF fed mice (p<0.0001). However, calorie consumption did not differ between WT and Plin2-null mice on either diet (Fig 1B). There also was no difference between LF and HF fed WT or Plin2-null mice in their energy expenditures (Fig 1C). Net energy balance data (Fig 1D) shows that on the LF diet both WT and Plin2-null mice were generally in energy balance, whereas both strains on the HF diet had a positive energy imbalance of approximately 8 Kcal/day. As expected because of the brief period of HF feeding, we did not detect differences in liver histological properties of LF or HF fed WT or Plin2-null mice nor did we detect evidence of lipid accumulation in any of the livers (Fig 1E). Despite similarities in energy balance and body compositions, we found that the respiratory exchange ratio (RER) values of WT were significantly greater than RER values for Plin2-null regardless of the diet composition (p = 0.03) (Fig 1E) in this acute feeding model. We did not detect difference between the activity levels of WT and Plin2-null mice on either diet, although WT and Plin2-null mice fed the HF diet had significantly (p<0.0005) lower overall activity than LF fed mice (Fig 1F).

### Plin2 affects intestinal neutral lipid properties

**Fig 2:**
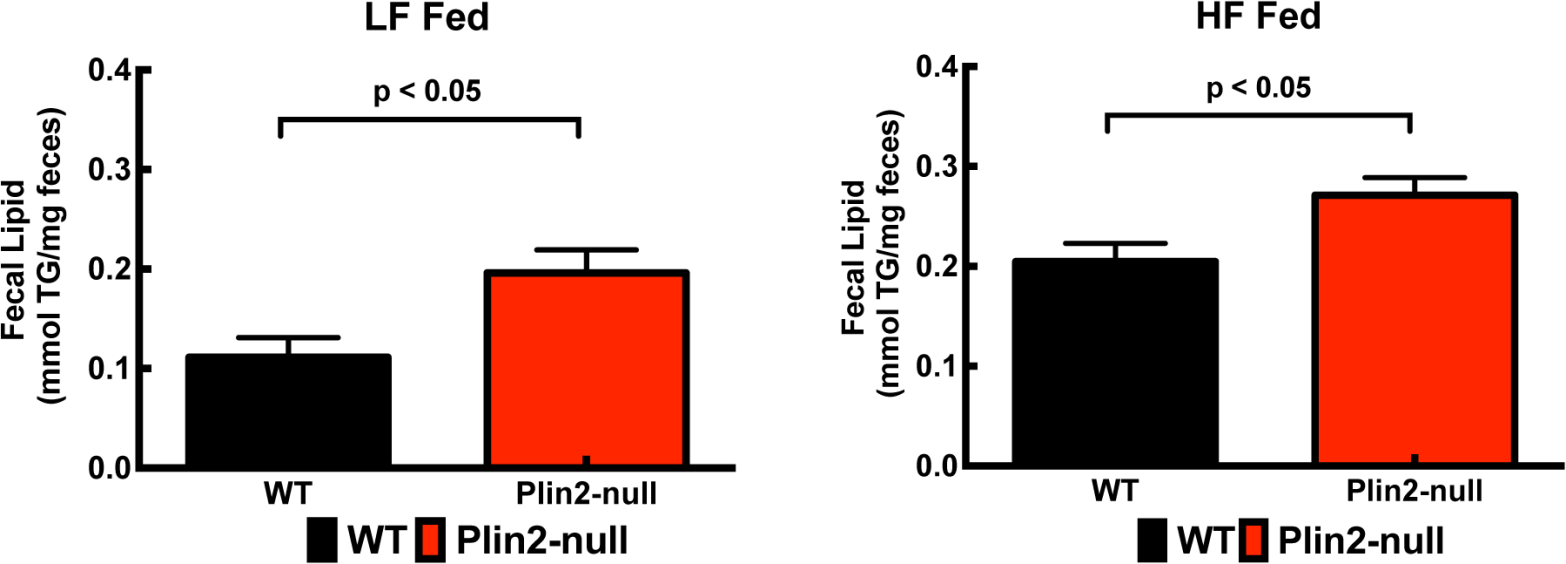
Effects of LF and HF diet feeding on fecal lipid contents. Fecal lipid quantities for WT and Plin2-null mice fed LF (A) or HF (B) diets for four days. All values are means ± SD, N = 4 per group. P-values were determined using the unpaired t-test (Prism 6, GraphPad Prism).

HF diet feeding is associated with altered intestinal function [53,54]. Plin2 is abundantly expressed in the mouse intestinal epithelium and is hypothesized to contribute to lipid uptake properties of the intestinal mucosa [15]. To determine if Plin2 deletion influences intestinal lipid absorption functions in response to short-term changes in dietary fat content, we quantified TG levels in feces of WT and Plin2-null mice after feeding LF or HF diets for four days. Fecal lipid contents of LF or HF fed WT mice were significantly lower than those of the corresponding Plin2-null mice (Fig 2A and 2B).

**Fig 3:**
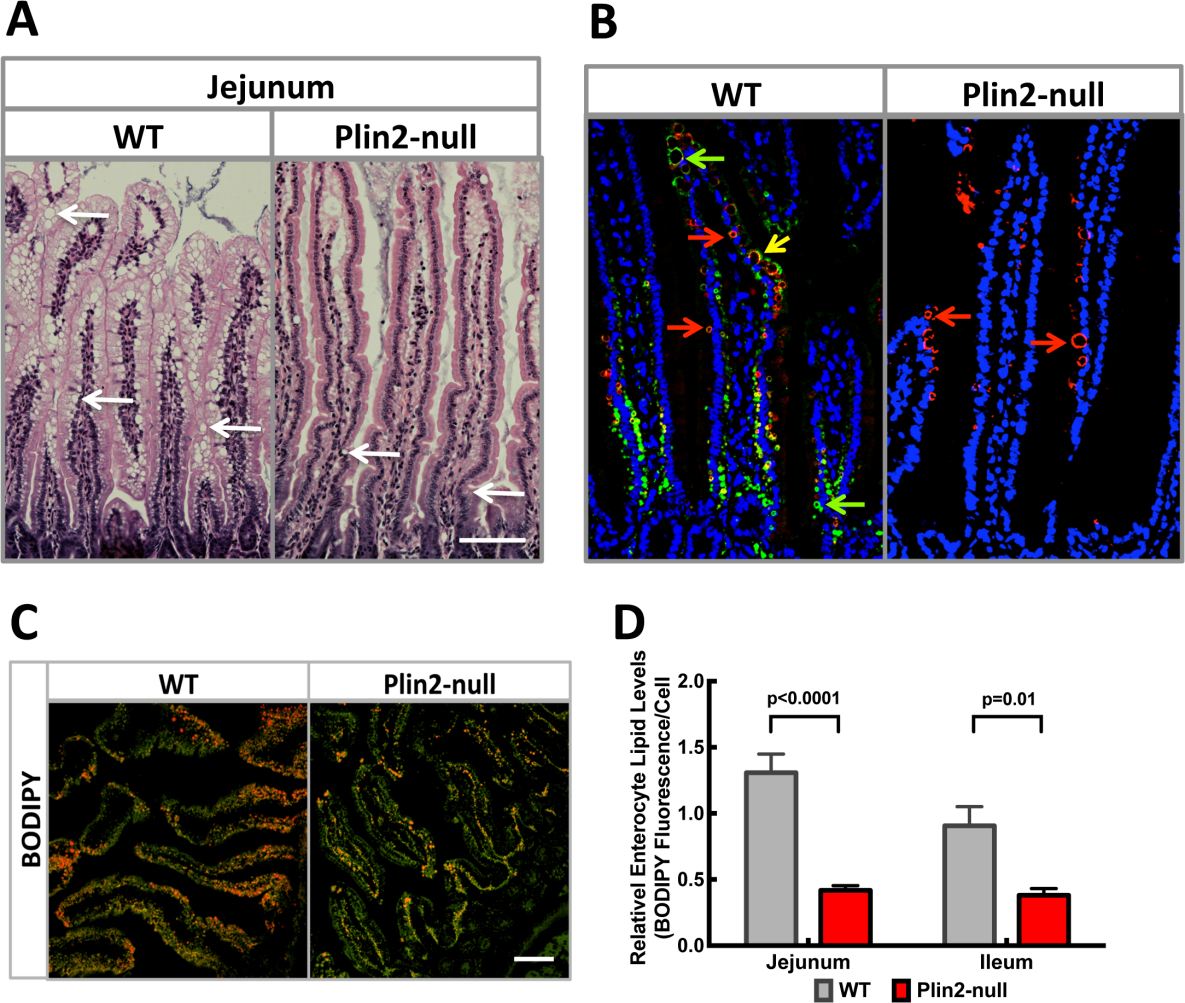
Effects of HF diet feeding on intestinal lipid properties. (A) Representative H&E stained jejunal sections from HF fed WT and Plin2-null mice. Note the apparent greater abundance of CLD (arrows) in intestinal villi from WT mice compared to Plin2-null mice. Scale bar = 100 μm. (B) Representative immunofluorescence images of jejunal sections from fasted and HF fed WT and Plin2-null mice stained with guinea pig anti-Plin2 (green) and rabbit anti-Plin3 (red) showing Plin2- (green arrows) and Plin3- (red arrows) coated CLD. Yellow arrow indicates CLD coated with Plin2 and Plin3. (C) BODIPY stained CLD (Red) and Alexafluor-WGA (green) stained intestinal villi surfaces from fasted and fed WT and Plin2-null mice. Scale bar = 100 μm. (D) Relative enterocyte lipid levels. Values are mean BODIPY fluorescence area/cell ± SEM from 8–10 200X sections from the jejunal and ileal regions of each mouse. Four animals per group were analyzed. P-values were determined using the unpaired t-test (Prism 6, GraphPad Prism).

The effects of Plin2 deletion on intestinal lipid properties were evaluated by comparing intestinal histology and intestinal lipid droplet staining properties in 4 day HF fed WT and Plin2-null mice that were fasted and then refed a HF diet. Significant numbers of large and small CLD were detected in enterocytes of H&E stained sections of WT mice. In contrast, only limited numbers of CLD were found in jejunal enterocytes of Plin2-null mice (Fig 3A). Immunofluorescence analysis documented that Plin2 and Plin3 coated CLD in WT enterocytes, and in some cases both Plin2 and Plin3 were found to coat the same lipid droplet (Fig 3B). As expected, enterocyte CLD in Plin2-null mice were positive for Plin3 but lacked Plin2 (Fig 3B). To quantify effects of Plin2 deletion on enterocyte lipid content, we stained intestinal sections from the jejunum and ilium of fasted and refed mice with BODIPY to label neutral lipids (Fig 3C) and calculated the relative level of fluorescence staining per cell (Fig 3D) [22]. The BODIPY fluorescence results indicate that neutral lipid levels in enterocytes in jejunum and ileum of WT mice are significantly greater than those of Plin2-null mice. Collectively, the results from quantitation of fecal and enterocyte lipid levels are consistent with the proposed role of Plin2 in regulating intestinal lipid absorption after high fat feeding [15], and provide evidence that Plin2 loss may impair this process. Moreover, the observation that Plin3 coats CLD in enterocytes of Plin2-null mice combined with the reduced numbers of BODIPY-stained CLD in jejunal and ileal enterocytes of Plin2-null mice, suggests that Plin3 may not fully compensate for Plin2 in regulating intestinal lipid properties.

### Plin2 deletion and dietary fat intake alter the gut microbiome

**Fig 4:**
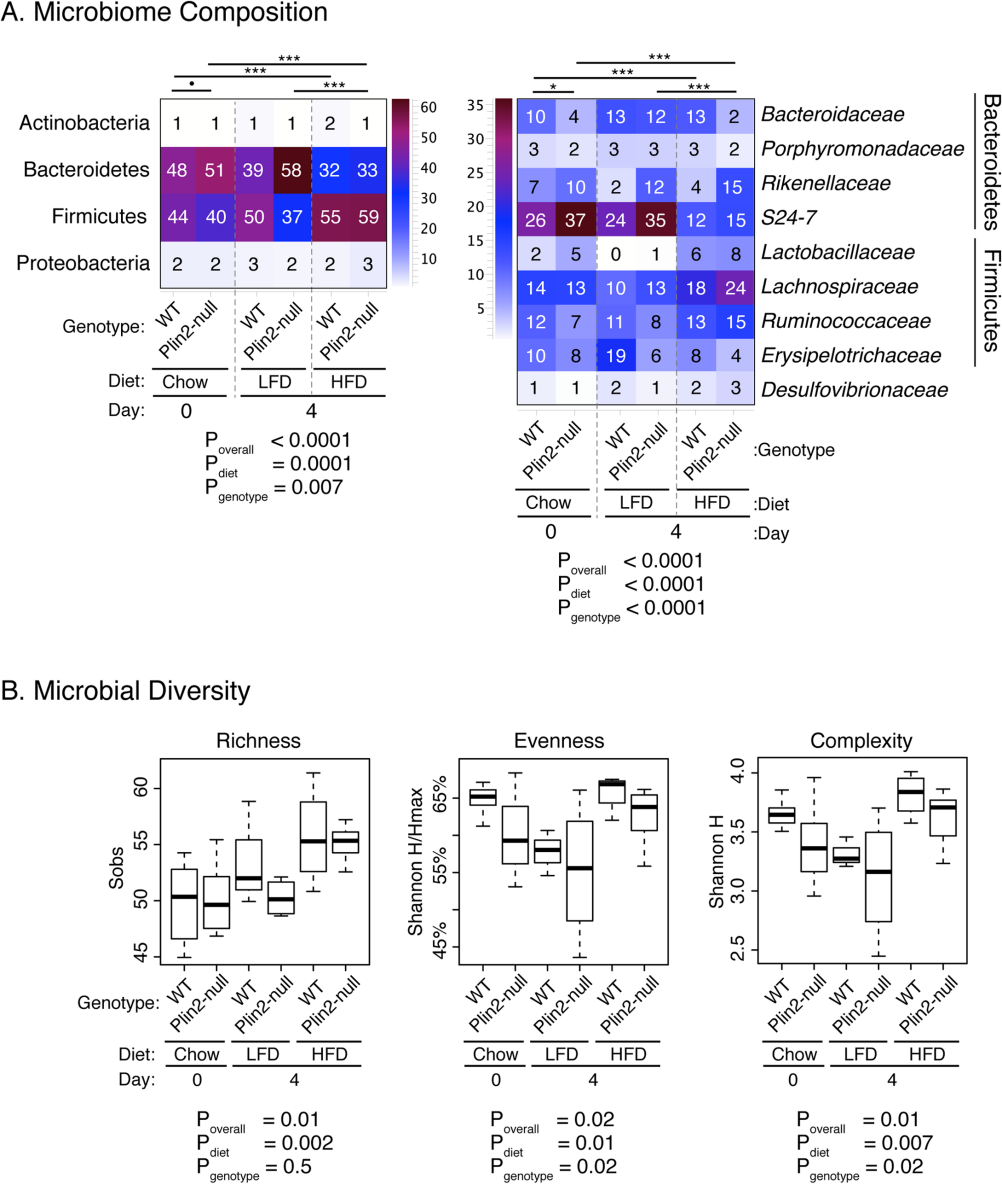
Microbiome composition and biodiversity are altered by genotype and diet. Fecal microbiomes from WT and Plin2-null mice on standard chow, low fat (LF), and high fat (HF) diets were profiled by high-throughput 16S rRNA amplicons sequencing. Panel A displays the distributions of bacteria among phyla (left) and family-level taxonomic categories (right). For simplicity, only taxa with mean relative abundances >2% are displayed; numerical values are the median within-group abundances of taxa. Overall p-values (P_overall_) were ascertained by non-parametric PERMANOVA tests for all six diet/genotype groups, using Morisita-Horn dissimilarity scores. PERMANOVA using both diet and genotype as predictor variables was performed to calculate adjusted p-values (P_diet_ and P_genotype_). Significant results of pairwise PERMANOVA tests performed on each pair of genotype/diet groups are indicated by horizontal bars above each heatmap: •: p<0.1; *: p<0.05; **: p<0.01; ***: p<0.001. Panel B displays the differences in biodiversity indices (richness, evenness, and complexity) by genotype and diet categories. Overall p-values (P_overall_) were ascertained by ANOVA tests for all six diet/genotype groups. ANOVA using both diet and genotype as predictor variables was performed to calculate adjusted p-values (P_diet_ and P_genotype_).

Because dietary fat content is a key regulator of gut microbiome composition [55], we next examined how altered intestinal lipid properties in Plin2-null animals affected the gut microbiome. Significant differences in fecal microbiome composition (p = 0.02 for non-parametric PERMANOVA of family-level data [36]) were evident between chow fed WT and Plin2-null mice at baseline (Day 0) prior to randomization to LF or HF diets (Fig 4A). At the phylum level, Plin2-null mice harbored significantly greater relative abundances (RA) of Bacteroidetes at baseline compared with WT mice (51.1% vs. 47.7% RA; p = 0.05), whereas Firmicutes trended to lower RA in Plin2-null mice (39.5% vs. 44.3% RA; p = 0.25). Within the phylum Bacteroidetes, Plin2-null animals were enriched in the uncultured family S24-7 (36.8% vs. 25.7% RA; p = 0.05), whereas the family *Bacteroidaceae* was reduced (3.6% vs. 10.0% RA; p = 0.05) compared with WT animals. Likewise within the phylum Firmicutes, the family *Ruminococcaceae* was present at diminished levels in Plin2-null mice (6.9% vs. 12.0%; p = 0.02).

Initiation of HF diet feeding resulted in significant, rapid changes in fecal microbiomes of both Plin2-null (p<0.001 for family-level PERMANOVA) and WT mice (p<0.001 for family-level PERMANOVA), compared to the respective, chow-fed mice at baseline. Furthermore, Plin2-null and WT animals on HF diet differed in fecal microbiomes (p = 0.09 for PERMANOVA). In both genetic backgrounds, HF feeding for 4 days resulted in loss of Bacteroidetes (Plin2-null: 51% vs. 33.4% RA for chow vs. HF, p = 0.004; WT: 47.7% vs. 31.8% RA, p = 0.009; Fig 4A) compared with chow-fed animals. The change in Bacteroidetes upon switching from chow to HF diet was largely attributable to loss of the family S24-7 in both Plin2-null (36.8% vs. 14.7% RA, p = 0.007) and WT (25.7% vs. 12.3% RA, p = 0.02) mice. The Bacteroidetes family *Rikenellaceae* (mainly the genus *Alistipes*) also was significantly reduced in WT mice (7.2% vs. 3.6% RA, p = 0.02) but was unaffected in Plin2-null mice (p = 0.52).

Loss of Bacteroidetes on HF diets was accompanied by significantly increased relative abundances of Firmicutes in both Plin2-null and WT strains (Plin2-null: 39.5% vs. 59.0% RA for chow vs. HF, p = 0.004; WT: 44.3% vs. 54.7% RA, p = 0.009; Fig 4A). Within the Plin2-null mice the increased Firmicutes arose primarily through expansion of the clostridial families *Lachnospiraceae* (13.2% vs. 23.6% RA, p = 0.18) and *Ruminococcaceae* (6.9% vs. 15.5% RA, p = 0.007). The Firmicutes families *Enterococcaceae* (p = 0.01), *Streptococcaceae* (p = 0.007), and *Peptococcaceae* (p = 0.03) also increased upon HF feeding, though these families accounted for a small fraction of the overall increase in Firmicutes RA. In contrast, among the WT mice the *Lactobacillaceae* were significantly increased (2.0% vs. 6.3% RA, p = 0.08), *Lachnospiraceae* trended towards increased densities (14.3% vs. 18.3%, p = 0.18), and *Ruminococcaceae* were unchanged in chow compared with HF feeding (12.0% vs. 12.7%, p = 0.48).

The fecal microbiomes of Plin2-null and WT animals on HF diet did not differ in composition between genotypes (p = 0.14 for family-level PERMANOVA) and no individual phyla or families were significantly different in abundance following FDR correction. However, trends toward reduced *Rikenellaceae* (3.6% vs. 14.9% RA, p = 0.13) and increased *Bacteroidaceae* (12.6% vs. 1.5% RA, p = 0.4) were noted in WT compared with Plin2-null mice.

Switching from chow to low fat (LF) diet feeding did not result in appreciable changes in the fecal microbiomes of Plin2-null (PERMANOVA p = 0.34 for Plin2-null mice on LF diet vs chow) or WT mice (PERMANOVA p = 0.6 for WT mice on LF diet vs chow). Nor did the microbiomes of Plin2-null and WT mice differ on LF diet (PERMANOVA p = 0.22). This latter finding was perhaps unexpected, given that statistically significant differences between the microbiomes of WT and Plin2-null mice (p = 0.02) were noted on chow diet. To further explore the effects of LF diet, we modeled the microbiome data from chow and LF diet-fed animals as a function of both diet and genotype (i.e., microbiome ~ diet*genotype). The results of the PERMANOVA analysis indicated that genotype (p = 0.0019), but not diet (p = 0.32), was significantly associated with microbiome composition in this subset of mice. In contrast, both diet and genotype were significant drivers of microbiome in chow vs. HF diet-fed animals. Thus in the short term, the low fat diet had minimal effects on the fecal microbiota.

Both diet and genotype (i.e., Plin2-null vs. WT) were independently associated with significant differences in fecal microbial diversity (Fig 4B). The HF diet significantly increased richness (i.e., OTU counts per sample) compared with both chow (p<0.01) and LF (p = 0.08) diets; no significant difference was observed between chow and LF diets. In contrast, the LF diet was associated with significant decreases in both the complexity and evenness of fecal microbiomes, compared with both chow (p = 0.09, p = 0.05, for complexity and evenness respectively) and HF diets (p = 0.01, p = 0.01, respectively). These results are consistent with the significantly higher abundances of the S24-7 family of Bacteroidetes observed in LF-fed animals. Similarly, the Plin2-null genotype was associated with significantly lower microbiome evenness (p = 0.02) and complexity (p = 0.02), relative to the WT genotype, again likely due to expansion of the S24-7 group in Plin2-null mice on chow and LF diets (Fig 4A).

**Fig 5:**
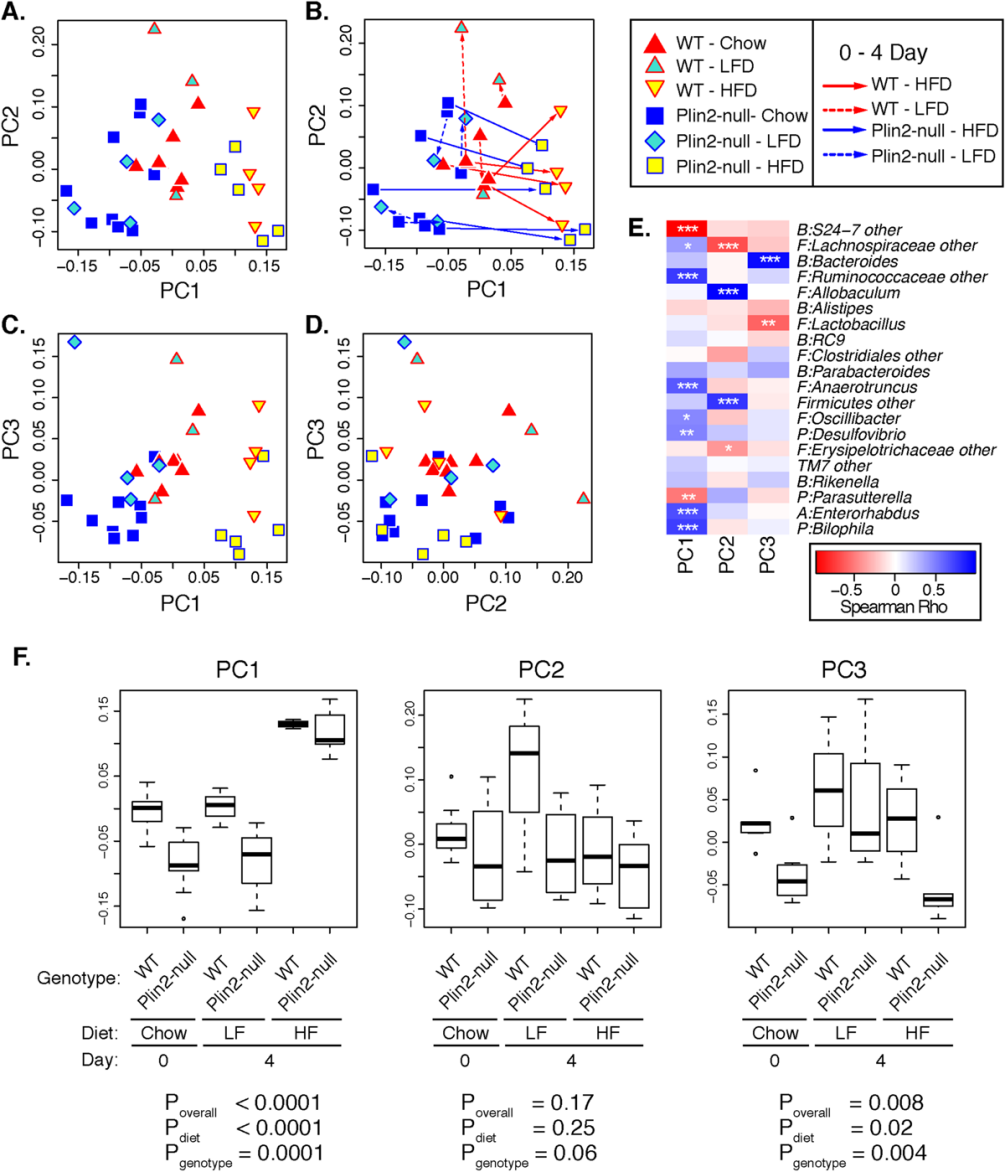
Principal components analysis of fecal microbiomes. PCA was carried out on genus-level microbiome datasets, as described in the text. PC1, PC2, and PC3 accounted for 35.3%, 26.8%, and 15.7% of the total variance in the dataset (77.8% cumulative proportion). Microbiomes from individual fecal samples, color-coded by genotype and diet, are plotted along the first three PC axes in Panels A, C, and D. Panel B displays the same plot as Panel A, but with arrows connecting pairs of samples from the same animals on different diets. Panel E displays a heat-map color-coding Spearman correlation coefficients between PC scores and the abundances of prevalent OTUs (abundances >1%) in fecal samples. OTU names are prefixed by letters indicating phyla classifications of the OTUs: A: Actinobacteria, B: Bacteroidetes, F: Firmicutes, P: Proteobacteria; T: TM7. Asterisks depict p-values for correlations: *: p<0.1; **: p<0.05; ***p<0.001. Blue shading indicates positive correlations, while red shading indicates negative correlations. OTUs that could not be classified to the genus level are labeled by adding “other” to the higher-order taxonomic assignment.

Data exploration through principal components analysis (PCA) indicated that both diet and genotype affected microbiome structure among the mouse groups (Fig 5; each symbol represents an individual mouse, color-coded by diet and genotype). Qualitatively, the HF diet appeared to have the strongest effects on sample clustering within the PCA plots, compared with the effects of either genotype or LF/chow diets, because all HF samples (regardless of genotype) segregated away from the remainder of the samples along PC1, PC2, and PC3 (lower right quadrants of Fig 5A, 5B and 5C). Indeed, PC1 scores were significantly associated with diet (p = 6.4e-11) and genotype (p = 0.0001), whereas PC2 and PC3 scores were weakly associated with genotype and diet (Fig 5F). Higher PC1 scores, which were associated with a HF diet (Fig 5F), were negatively correlated with the Bacteroidetes family S24-7 and positively correlated with several groups of Firmicutes, Actinobacteria, and Proteobacteria (Fig 5E). Similarly, higher PC3 scores were observed for WT mice, compared with Plin2-null mice, and were positively correlated with the genus *Bacteroides*. Thus, differential shifts in Firmicutes and sub-groups of Bacteroidetes (e.g. S24-7 vs. *Bacteroides*) resulting from genotype and/or dietary factors help to explain the observed PCA clustering.

### Effects of Plin2-null and diet on fecal bacterial metagenomes

Because 16S profiling provides only limited information about functional differences between microbiomes, we also subjected fecal DNA samples from the Plin2-null and WT mice fed HF or LF diets to shotgun metagenomic sequencing on the Illumina MiSeq platform, generating 2x300 nt paired-end reads. A median of 4.6x10^5^ (IQR 3.7x10^5^, 8.4x10^5^) sequences were analyzed per sample using the web-based MG-RAST automated gene annotation pipeline [46].

**Fig 6:**
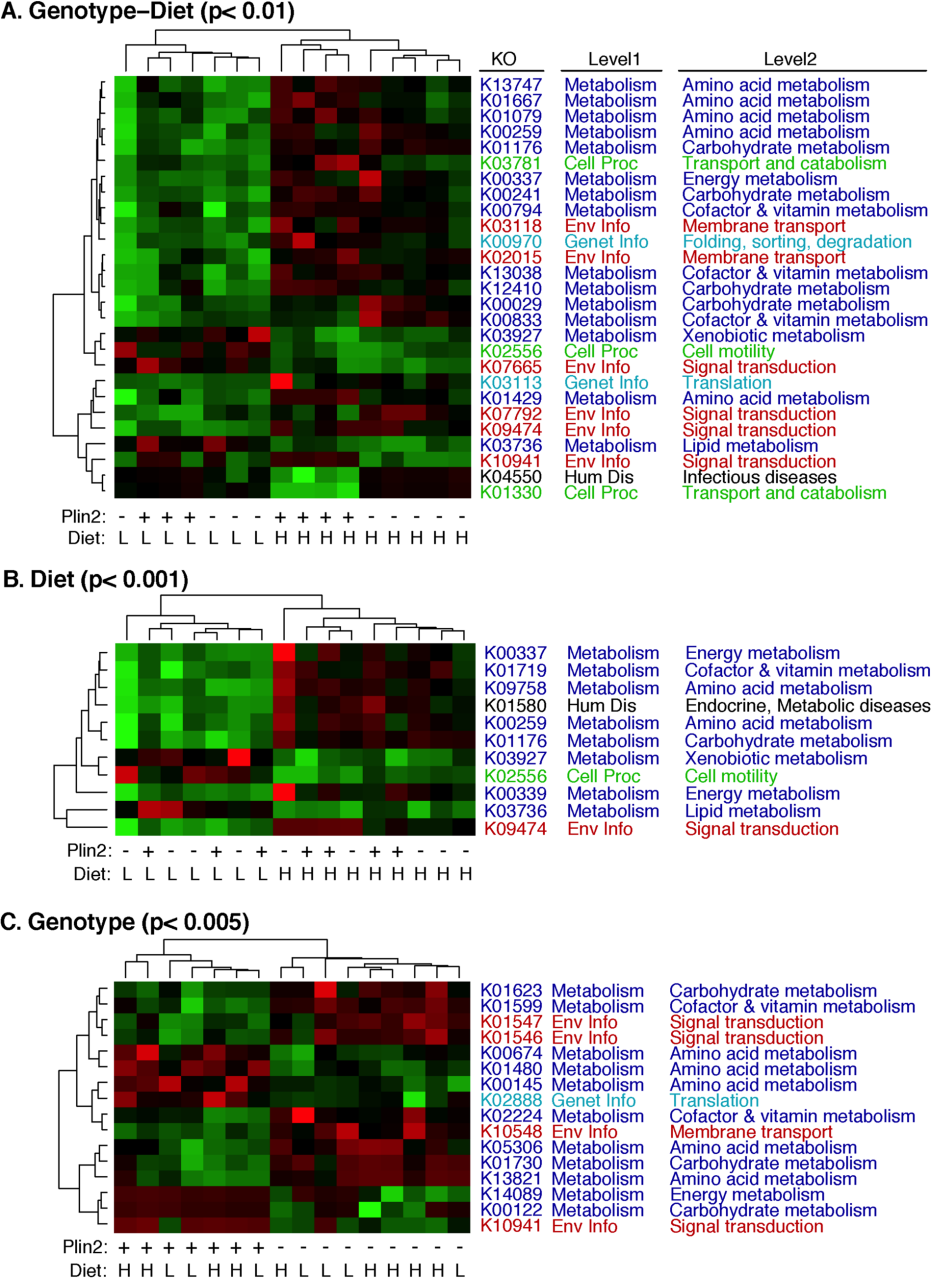
Influence of dietary fat and Plin2 genotype on fecal metagenomes. Metagenomic DNA reads were annotated against the KEGG orthology hierarchy [74] using MG-RAST[46] and significant KO categories identified as described in the text. The heatmaps display the normalized relative abundances of KO categories that varied significantly as a function of genotype combined with diet (Panel A), diet alone (Panel B), or genotype alone (Panel C). Unadjusted p-value cutoffs for each panel were chosen in order to display a representative selection of the data in a single figure.

A total of 3874 KEGG ortholog (KO) functional classes were identified at least once in the combined metagenomic dataset. Of these, 27 KO categories differed significantly (p<0.01) when both diet (HF vs. LF) and genotype (Plin2-null vs. WT) were combined into one predictor variable for Kruskal-Wallis tests (Fig 6A). Hierarchical clustering of animals based on KO frequency patterns among significant KO categories indicated that diet was a larger factor than genotype in determining fecal metagenome composition (Fig 6, Panel A). Regardless of genotype, HF and LF diets resulted in significant differences in the frequencies of multiple functional categories, which belonged primarily to the higher-order KO categories of “Metabolism”, “Cellular Processes”, and “Environmental Information Processing” (Fig 6A and 6B). A variety of metabolic functions, including those of amino acid, carbohydrate, cofactor, and xenobiotic metabolism, were identified through this analysis. Interestingly, only one KO, K03736 (ethanolamine ammonia-lyase small subunit [EC:4.3.1.7] belonging to the glycerophospholipid metabolism pathway ko00564), was associated with lipid metabolism. Nevertheless, 16 KO categories were found to differ significantly between Plin2-null and WT animals. Again, these genes were associated primarily with carbohydrate and amino acid metabolism, along with environmental sensing (Fig 6A and 6C).

## Discussion

Despite much concern among the public and biomedical community, obesity and its related co-morbidities continue to rise in global prevalence in both the pediatric and adult populations. Non-alcoholic fatty liver disease (NAFLD), an obesity-associated metabolic disorder, is one of the most prevalent causes of chronic liver disease and currently is the third leading cause of liver transplantation. Multiple lines of evidence implicate imbalances in lipid homeostasis as determinants of NAFLD pathogenesis. However, the molecular mechanisms linking disrupted lipid homeostasis to obesity and NAFLD remain to be fully described. We recently demonstrated that deletion of the Plin2 gene, largely abrogates the long-term effects of high fat (HF) diet induced obesity, insulin resistance, dyslipidemia, adipose inflammation, and NAFLD in mice. In the present study, we have examined the acute effects of 4-day exposure to HF or LF diet on murine growth properties, enterocyte lipid metabolism, and gut microbiome dynamics.

Plin2-null and WT mice did not differ from each other in body weight or their general metabolic properties on a standard chow diet. At the end of the 4-day feeding experiment, both WT and Plin2-null mice on HF diets exhibited comparable levels of energy intake, energy expenditure and activity, along with similar increases in body weight. However, Plin2-null mice were characterized by significantly lower RER values indicative of increased lipid oxidation [20] (Fig 1G), diminished enterocyte CLD content (Fig 3), and increased fecal triglyceride levels (Fig 2). Overall, these results suggest that Plin2 contributes to acute responses of lipid metabolism and possibly intestinal lipid absorption and/or secretion to alterations in dietary fat content. Interestingly, Plin2 deletion did not affect RER and fecal lipid levels in mice fed a HF diet for 12 weeks [13], thus suggesting that chronic HF diet feeding results in adaptations in lipid metabolism and dynamics of intestinal lipid responses of Plin2-null mice.

The results of the microbiome analysis, which employed both 16S rRNA profiling and metagenomic deep sequencing, indicated that both dietary fat content and Plin2 genotype were significantly and independently associated with gut microbiome composition, diversity, and function. Even at baseline, when fed identical, standard chow diets, significant differences in fecal microbiota were evident between Plin2-null and WT mice (Figs 4A, 4B and 5F). These differences were most evident in the higher abundances of Firmicutes and correspondingly lower abundances of Bacteroidetes (mainly the uncultured family S24-7) that were observed in WT compared with Plin2-null animals. An increased ratio of Firmicutes to Bacteroidetes has been associated with obesity and liver disease in some [6,56–58], but not all, [59–61] studies of obese humans and rodent models (reviewed in [55,62–64]. Therefore, even before the dietary intervention began, the Plin2-null mice carried a gut microbiome that was putatively less obesigenic than the microbiota of WT animals. Why significant microbiome differences observed between WT and Plin2-null mice on chow diets were not maintained when animals were switched to the LF diet is not clear. It is possible that this transition resulted in greater inter-animal variability, which obscured differences. Alternatively, the LF diet transition may have made contributions to gut microbiome properties in both WT and Plin2-null mice that resulted in relative normalization of their gut microbiomes.

On the HF diet, both WT and Plin2-null mice rapidly developed altered intestinal microbiomes, with 16S rRNA profiles that were characterized by significantly greater Firmicutes to Bacteroidetes ratios, compared with animals on chow or LF diets. The rapid loss of the Bacteroidetes family S24-7 (a clade related to the *Porphyromonadaceae*) upon initiation of HF diet feeding was particularly striking (Fig 4B). However, because the S24-7 family is relatively poorly characterized from a functional standpoint, little can be concluded about the particular contribution of this clade to host metabolism. Metagenomic sequencing of fecal DNA specimens also revealed multiple functional categories that were differentially present in HF- versus LF-fed animals (Fig 6A and 6B), including genes involved in diverse metabolic processes (e.g., amino acid, carbohydrate, cofactor, and xenobiotic metabolism) and environmental sensing. These results suggest that the coding capacity of the gut microbiome can rapidly respond (< 4 days) to shifts in the nutrient/metabolite microenvironment of the gut resulting from changes in dietary fat content. Similarly rapid changes have been observed in human subjects randomized to plant-based or animal-based diets, which induce diet-specific alterations in fecal microbiota [65].

As with diet, Plin2 genotype also was associated with altered abundances of multiple microbial genes involved in metabolism and environmental sensing (Fig 6A and 6C). We hypothesize that differences in the quantities and species of lipids present in the intestinal lumens of the WT and Plin2-null animals selected for particular fecal microbiotas and associated metagenomes. Interestingly, although the microbiomes of HF-fed Plin2-null and WT animals were comparable in terms of 16S rRNA profiles, metagenomic analysis revealed several KO classes (e.g. K10941, K04550, K01330; Fig 5A) that were dissimilar in abundance between these two groups. Further exploration of how these functional classes modify host physiology, especially lipid homeostasis, may help to explain why Plin2-null mice are protected from the long-term pathological effects of HF diets, despite bearing the hallmarks of obesigenic microbiomes (i.e., higher Firmicutes, lower Bactroidetes).

An inverse correlation between carbohydrate oxidation and the relative ratio of Bacteriodetes and Firmicutes in feces of human subjects [66] implicates contributions of gut microorganism metabolism to host fuel utilization. Consistent with this possibility, we found significant, energy balance independent decreases in RER values for Plin2-null relative to WT mice on either LF or HF diets. These values corresponded with increases in RA levels for members of the Bacteriodetes family Rikenellacea. Although additional studies are needed to establish that Plin2-dependent effects on microbiome composition directly contribute to changes in host metabolism, our data provide evidence that susceptibility to obesigenic diets may involve coordinate interactions between host lipid homeostasis mechanisms and specific gut bacterial populations that influence host fuel utilization.

Several limitations to this study must be noted. First, examination of the microbiome in fecal specimens, rather than those of the small intestine, cecum, or colon, may have overlooked significant, but localized alterations in the gut microenvironment arising from Plin2 genotype or dietary manipulation. However, our preliminary analysis of cecal contents corroborates the microbiome findings from fecal specimens. Second, the presence of a microbial gene in a metagenomic sequence dataset does not necessarily indicate its expression in the gut environment; follow up experiments using RT-QPCR or high-throughput RNA sequencing are needed to test differential gene expression in these experimental groups. Finally, gut-liver interactions involving the actions of bile acid, immune, and hormonal mediators are increasing recognized to be important influences intestinal function and gut microbiome properties [67–70]. In addition to its proposed roles in regulating hepatic and intestinal lipid properties [13,15,71], Plin2 has been implicated in the regulation of immune function and bile acid actions [72,73]. Thus, the effects of whole body Plin2 deletion on intestinal lipid properties and gut microbiome responses to dietary alterations potentially involve multiple organs and cell types and additional studies are needed to understand the extent to which intestinal Plin2 specifically contributes to diet-induced alterations gut microbial function.

In summary, our data suggest that Plin2 coordinates lipid homeostasis in the intestine, and that its effects on lipid uptake and transport by enterocytes modulate the capacity of the intestinal microbiome to contribute to diet-induced obesity and NAFLD. Future work will be directed towards gaining more mechanistic understanding of how microbiome-host crosstalk along the gastrointestinal mucosa leads to these increasingly prevalent and costly diseases.
